# Donations Made and Received: A Study of Disclosure Practices of Pharmaceutical Companies and Patient Groups in Canada

**DOI:** 10.34172/ijhpm.2021.172

**Published:** 2021-12-14

**Authors:** Joel Lexchin

**Affiliations:** ^1^School of Health Policy and Management, York University, Toronto, ON, Canada.; ^2^University Health Network, Toronto, ON, Canada.; ^3^Faculty of Medicine, University of Toronto, Toronto, ON, Canada.

**Keywords:** Canada, Donations, Patient Groups, Pharmaceutical Companies, Reporting

## Abstract

Given the increasing role of patient groups in pharmaceutical policy-making in Canada, this observational study was undertaken to determine whether companies that are members of Innovative Medicines Canada (IMC) list, on their publicly available websites, the names of patient groups that they make donations to and reciprocally, whether patient groups publicly list the names of the companies that they receive donations from. Websites of IMC members were searched for the names of the patient groups receiving donations, value of the donations and year the donations were made. The website of each patient group that was listed as receiving a donation was then searched for information about the name of companies making donations along with the value of the donations, year the donations were made and percent of the patient groups’ income represented by the donation. For donations over $50 000, an attempt was made to match donations that companies made to donations that patient groups received. Eleven of 44 IMC members reported making 165 donations to 114 different patient groups. Seventy-nine of these 114 groups reported receiving 373 donations from IMC members. Information about the value of donations, the year that they were given and received and the percent of patient groups’ income that they represented was limited. Donations made and received could not be matched because of the absence of information about the donations. Reporting on websites about donations by both companies and patient groups in Canada is haphazard, inconsistent and incomplete. Reforms are need to both the way that companies and patient groups report donations.

## Introduction

 Understanding financial relationships between patient groups and pharmaceutical companies is a necessary component of evaluating the position that patient groups take when the interests of companies are involved. One example of where patient groups may be favouring the companies that are donating to them is with respect to the submissions that they make to the Common Drug Review (CDR) and the panCanadian Oncology Drug Review (pCODR), both arms of the Canadian Agency for Drugs and Technology in Health. CDR and pCODR conduct health technology assessments and make recommendations to public drug plans (except the one in Quebec) about whether the provincial drug plans should fund medications for particular indications. When patient groups make submissions, they need to declare any financial conflicts of interest (ie, grants, donations and other transfers of value) they have with pharmaceutical companies and these conflicts are made publicly available. Up until July 22, 2018, 93 patient groups made 372 submissions and declared conflicts in 324 (87%) of them and the groups supported funding in over 90% of their submissions.^1^

 Internationally, reporting of donations by large companies is variable^2^ but this issue has not been investigated in the Canadian context. Since 2009, the Code of Ethical Practices from Innovative Medicines Canada (IMC), the lobby group representing brand-name manufacturers, has included a voluntary guideline for its membership stating that they should “disclose, by means of their web sites and annual reports, a list of all stakeholders to which they provide direct funding.”^3^ However, there are no penalties for not disclosing and no evaluation has been undertaken to determine if companies are voluntarily complying with this provision. There are also no voluntary or mandatory requirements for companies that are not IMC members to report donations (IMC members are the largest component of the research-based industry in Canada, accounting for 73% of the dollar sales of patented medicines^4^).

 In Canada, it is difficult to obtain information on funding of patient groups by pharmaceutical companies aside from what is revealed in submissions to CDR and pCODR. Most patient groups are registered charities and file annual financial reports to the Canada Revenue Agency, but those publicly available reports do not contain information about individual donations. See, for example, the financial report for Canadian Cancer Survivor Network at https://apps.cra-arc.gc.ca/ebci/hacc/srch/pub/dsplyRprtngPrd?q.srchNm=canadian+cancer+survivor+network&q.stts=0007&selectedCharityBn=834540882RR0001&dsrdPg=1. The question about whether patient groups voluntarily reveal the names of pharmaceutical company donors and information about the donations on their websites has not been investigated.

 This study was undertaken to determine whether companies that are members of IMC list on their publicly available websites the names of patient groups that they make donations to and what other information about the donations is provided. A similar search of the websites of the named patient groups was done to see if they publicly list the names of the companies that they receive donations from and what other information they provide about the donations. The study also investigates the extent of congruency between company and patient group disclosures of donations made and received.

## Methods

###  Source of Data About Donations

 The websites of all 44 IMC members^5^ were manually searched between March 24-26, 2021 to determine if the company listed patient groups that it made donations to. If the manual search was unsuccessful, then a Google search of the website was done using the terms “donation,” “grant,” “patient group” and “sponsor.” If names of patient groups were found, then the name of each patient group was entered into an Excel spreadsheet along with the URL for the company webpage containing the names, the pathway and number of mouse clicks from the website home page to the page containing the information about donations, the year donations were made, the value of donations in dollars and the purpose of donations. When it was unclear if different names referred to the same patient group, a Google search was done on all the names. If the search returned the same URL for each name, then the conclusion was reached that it was a single group. If a company made a donation to multiple branches of the same organization each donation was treated separately, eg, donations by a single company to Lung Association Canada, Lung Association – British Columbia and Lung Association – Manitoba were treated as three separate donations. If a single company made multiple donations to one group in the same year, it was treated as a single donation.

 Patient groups were defined as those whose primary mission is to combat a particular disease or disability or to work toward improving the health and well-being of a particular patient population.^6^ In-line with the definition of a patient group, donations to other organizations, for example, foundations whose primary focus was on fund raising for a particular disease, coalitions of patient groups focusing on diverse diseases or to non-Canadian groups were not included. International groups with a branch in Canada were only considered if it was clear that the Canadian branch received funding directly from one or more pharmaceutical companies and made independent decisions about how the donations were spent. (No organization met this definition).

 The website of each patient group that was listed as receiving a donation was then manually searched, also between March 24-26, 2021, to determine if the group disclosed the names of the donor companies and other information about the donation. If the manual search was unsuccessful, then a Google search of the website was done using the name(s) of one or more companies that declared making a donation to the group. If the names of companies were found, then the following data were collected and entered into an Excel spreadsheet: name(s) of IMC member companies making the donations, URL for the webpage containing the names, pathway and number of mouse clicks from the website home page to page containing the information about the donation, year the donations were received, value of the donations in dollars, purpose of the donations and percent of group’s income represented by the donations. If patient groups listed the names of donors and information about donations in multiple places on their websites, eg, in their annual reports and elsewhere on their websites, then the most comprehensive information was used. Donations to a single group from a parent company and one of its subsidiaries were treated as coming from different companies if the parent and subsidiary were both members of IMC. In addition, donations to patient groups from non-IMC members were recorded.

 All dollar amounts are reported in Canadian dollars.

 Patient groups and companies were not directly contacted as the study aim was to assess the completeness of available information on donations on public websites.

###  Analysis

 Counts were done of the number of companies that reported making donations, the number of donations per company and whether the following information was available: year of the donation, value of donation and purpose of the donation. The same information was summed for donations that patient groups reported receiving, plus information about the percent of the group’s income represented by donations from companies. Only descriptive data is reported. This study followed the Strengthening the Reporting of Observational Studies in Epidemiology (STROBE) reporting guideline for cross-sectional studies.^7^

###  Ethics and Data Availability

 Since all information was publicly available ethics approval was not required. The datasets collected and analyzed during the current study are available from the corresponding author on reasonable request.

## Results

###  Reporting of Donations by Companies

 Eleven of the 44 members of IMC reported on their websites making 165 donations to 114 patient groups (Table 1). (Supplementary file 1 lists companies not reporting making donations). Individual companies made donations to between 1 and 46 different patient groups. The median number of companies reporting donations to a single group was 1 (interquartile range [IQR] 1, 2), but in one case 5 companies reported donating to a single patient group. Six companies stated the year the donation was made – one said 2018, two said 2019 and the other three said 2020. Six companies (AbbVie, Leo, Novo Nordisk, Paladin, Purdue, Takeda) did not disclose the dollar amount of any of the donations that they made. Bayer stated that all donations were for greater than $5000. Paladin disclosed the purpose of the single donation listed on its website. Pfizer disclosed the amount and purpose of the single donation that was reported on its website, while Roche, GlaxoSmithKline and Novartis did the same for their multiple donations.

**Table 1:** Innovative Medicines Canada Member Companies Reporting Making Donations to Patient Groups

**Name of Company**	**No. of Patient Groups That Received Donations**	**Year Donations Made**	**Amount of Donation **	**Purpose of Donation **	**Pathway From Home Page of Company to List of Patient Groups Receiving Donations (No. of Mouse Clicks)**
AbbVie	45	Not stated	Not stated	Not stated	Community: Patient Associations (2)
Bayer	16	2020	Greater than $5000	Not stated	This is Bayer: Our Business Areas: Pharmaceuticals: Grants and Sponsorships (4)
GlaxoSmithKline	16	2020	Exact amount	Yes	Responsibility: Responsibility reports and additional data: Patient group funding (3)
Roche	24	2019	Exact amount	Yes	Menu: About Roche: Ethics and integrity: Patient organization grants and donations (leaving Roche Canada website): Working with patient organizations (4)
Leo	3	Not stated	Not stated	Not stated	About us: Corporate social responsibility (2)
Novartis	46	2019	Exact amount	Yes	About us: Corporate responsibility: The Novartis commitment to patients and caregivers: Patient community (leaving Novartis.ca website): Our company: Corporate responsibility: Corporate responsibility reporting and disclosure: Transparency and disclosure: Patient organization funding: Download the 2019 report (10)
Novo Nordisk	7	Not stated	Not stated	Not stated	About us: In the community (2)
Paladin	1	2020	Not stated	Yes	Community (1)
Pfizer	1	2018	Exact amount	Yes	Media centre: Pfizer Canada announces support to Heart & Stroke's #timetoseered campaign (2)
Purdue	3	2018	Not stated	Not stated	Corporate social responsibility: Sponsors, donations and grants: Grant recipients: Grant recipient list 2018 (4)
Takeda	3	Not stated	Not stated	Not stated	Who we are: Our partnerships (2)

###  Placement of Information About Donations on Company Websites

 Donations were listed in multiple places on companies’ websites and it took a median of 2 mouse clicks (IQR 2, 4) to find the information. For Paladin it took only a single mouse click from the home page to access the information about the donation, whereas for Roche and Novartis it required leaving their Canadian webpages and reaching the information for Novartis took 10 mouse clicks (Table 1).

###  Reporting of Donations by Patient Groups

 Seventy-nine of 114 (69.3%) patient groups reported receiving a total of 373 donations (median number 4, IQR 2, 7) (Table 2 and Supplementary file 2), including from 19 of the 33 IMC members that did not report making donations on their websites. Thirty-five out of 114 patient groups (30.7%) that companies reported making donations to did not acknowledge receipt of donations from that company. (Supplementary file 3 lists the patient groups not reporting receiving a donation from IMC member companies). Eighteen of the 79 groups that reported receiving donations did not list all the companies that claimed that they donated to the groups. Janssen and Merck did not report making any donations and Pfizer only reported a single donation. Summing the number of patient groups that reported receiving donations from one or more of these three companies, each of the companies made donations to more than 25 different groups that they did not report.

**Table 2:** Information About Donations Reported by Patient Groups

**No. of Patient Groups Reporting Receiving Donations **	**Median Number of Donations Received Per Group (IQR)**	**No. of Groups Reporting Year Donation Received (Percent of Total)**	**No. of Groups Reporting Value of Donations**			**No. of Groups Reporting Percent of Overall Income From Donations (Percent of Total)**	**No. of Group Reporting Purpose of Donations (Percent of Total)**	**Median No. of Mouse Clicks to Find Information About Donation (IQR)**
			**Dollar Range**	**Category** ^a^	**No Information**			
79	4.0 (2.0, 7.0)	20 (25.3)	14 (17.7)	8 (10.1)	57 (72.2)	0 (0.0)	4 (5.1)	2.0 (1.0, 2.0)

Abbreviation: IQR, interquartile range. ^a^ Category, eg, gold, silver, bronze donation.

 Twenty patient groups (25.3% of all groups reporting any donations) stated the year when they received the donations; aside from two groups that reported receiving donations in 2017, all the other groups said the donations were received in 2019 or after (Table 2 and Supplementary file 2). Fourteen groups (17.7%) reported the dollar range of the donations (eg, $5000-$10 000), 8 groups (10.1%) put donations into categories (eg, gold, silver, bronze) without giving a dollar value for the categories and the remaining 57 groups (72.2%) did not report the value of the donations. Four groups provided information about the purpose of donations, but none of the 79 groups that received donations said the proportion of their overall income represented by the donations or gave information that would allow this calculation. Eight patient groups also reported receiving donations from IMC itself. The IMC website did not report any of these donations. Finally, 47 patient groups reported the receipt of 95 donations (1 to 11 donations per group) from 56 non-IMC member companies.

###  Placement of Information About Donations on Patient Group Websites

 Locating information about the donations required a median of 2 mouse clicks (IQR 1, 2). Fourteen groups listed the names of company donors on their website home page and 40 used the pathway starting with “About us” or the equivalent. But even within this latter group there was variability; usually the names were under a heading such as “Sponsors” or “Partners” but sometimes they were only in the annual reports. The pathways in other groups were less obvious, for instance Muscular Dystrophy Canada’s list of donors was in: Services & support: Research: Research new: Key topics in Spinal Muscular Atrophy research discussed at first ever Muscular Dystrophy Canada SMA Research Summit; for Rethink Breast Cancer the list was in: Support our movement: Partner with us (Table 2).

###  Congruence Between Companies Reporting Donations and Patient Groups Acknowledging Receipt of Donations

 Congruence between companies reporting donations and patient groups acknowledging receipt of these donations might be more likely for large donations. Table 3 examines donations (≥$50 000) to test this hypothesis. In a few cases the difference in dates between when the donation was made and received probably meant that it was not the same donation, but in general it was not possible to establish a correlation because even when patient groups reported receiving a donation from the company they often did not give the amount of the donation or the year when it was received.

**Table 3:** Donations $50 000 and Greater Listed on Innovative Medicines Canada Member Companies’ Websites and Patient Groups’ Acknowledgement of Receipt of Donations

**Company Making Donation**	**Amount **	**Year Company Said Donation Given**	**Patient Group Receiving Donation**	**Patient Group Acknowledges Receiving a Donation From Same Company on Group’s Website**	**Year Patient Group Said Donation Received**
GlaxoSmithKline	$50 000	2020	Lung Association – British Columbia	No	Not stated
GlaxoSmithKline	$139 230	2020	Lung Association - Quebec	GlaxoSmithKline listed as “gold” partner	Not stated
GlaxoSmithKline	$67 500	2020	Lung Health Foundation	GlaxoSmithKline listed as donor of $100 000-$199 999	2019-2020
GlaxoSmithKline	$140 000	2020	Ovarian Cancer Canada	No	Not stated
Novartis	$50 000	2019	Arthritis Consumer Experts	No	Not stated
Novartis	$102 000	2019	Arthritis Society	Yes	Not stated
Novartis	$66 870.28	2019	Canadian Breast Cancer Network	Yes	Not stated
Novartis	$94 502.39	2019	Canadian Council for the Blind	Novartis listed as “gold” partner	2021
Novartis	$53 216.18	2019	Canadian MPN Network	No	Not stated
Novartis	$53 820	2019	Canadian National Institute for the Blind	Novartis listed as donor of $25 000-$99 999	2020-2021
Novartis	$95 000	2019	CNETS	Yes	2020
Novartis	$89 046.79	2019	Foundation Fighting Blindness	Yes	Not stated
Novartis	$60 000	2019	Leukemia & Lymphoma Society of Canada	No	Not stated
Novartis	$50 000	2019	Lymphoma Canada	Yes	Not stated
Novartis	$50 000	2019	Migraine Canada	Yes	Not stated
Novartis	$130 250	2019	MS Society of Canada	No	Not stated
Novartis	$71 125	2019	Rethink Breast Cancer	No	Not stated
Novartis	$61 790.11	2019	Save Your Skin Foundation	Yes	Not stated
Pfizer	$100 000	2018	Heart & Stroke	Pfizer listed as donor of $25 000-$49 999	Not stated
Roche	$80 000	2019	Canadian Breast Cancer Network	Roche listed as donor but amount not given	Not stated
Roche	$210 222	2019	Canadian Hemophilia Society	Roche listed as donor of >$10 000	Not stated
Roche	$71 612	2019	Huntington Society of Canada	Roche listed as donor of $100 000-$249 999	2019
Roche	$60 000	2019	Lung Association - Quebec	No	Not stated
Roche	$75 000	2019	Lung Cancer Canada	Roche listed as donor but amount not given	2019
Roche	$100 000	2019	Lymphoma Canada	Roche listed as donor but amount not given	Not stated
Roche	$126 900	2019	MS Society of Canada	No	Not stated
Roche	$60 000	2019	Rethink Breast Cancer	Roche listed as donor but amount not given	Not stated

Abbreviations: CNETS, Canadian Neuroendocrine Tumour Society; MS, multiple sclerosis.

###  Congruence Between Patient Groups Reporting Receipt of Donations and Companies Acknowledging Making Donations

Table 4 is the reciprocal of Table 3 and shows that when patient groups list donations of $50 000 or more on their websites it is very difficult to establish congruence between donations that patient groups report receiving and companies report making because of the lack of details about the exact value of the donation and the date when it was received and made.

**Table 4:** Donations $50 000 and Greater Listed on Patient Groups’ Websites and Companies’ Acknowledgement of Making the Donations

**Patient Group Receiving Donation**	**Amount**	**Year Patient Group Said Donation Received**	**Company Making Donation**	**Company Acknowledges Making a Donation to Same Patient Group on Company’s Website**	**Year Company Said Donation Made**
Canadian Cancer Society	$50 000-$99 999	Not stated	AstraZeneca	No	Not stated
Canadian Cancer Society	$>100 000	Not stated	Janssen (Johnson & Johnson)	No	Not stated
CATIE	>$70 000	Not stated	Gilead	No	Not stated
Diabetes Canada	$150 000-$399 999	Not stated	AstraZeneca	No	Not stated
Diabetes Canada	$50 000-$149 999	Not stated	Lilly	No	Not stated
Diabetes Canada	$150 000-$399 999	Not stated	Janssen	No	Not stated
Diabetes Canada	>$400 000	Not stated	Novo Nordisk	Diabetes Canada listed as recipient of grant, amount not given	Not stated
Diabetes Canada	$50 000-$149 999	Not stated	Sanofi	No	Not stated
Heart & Stroke Foundation	$50 000-$99 999	Not stated	Bayer	No	2020
Heart & Stroke Foundation	$50 000-$99 999	Not stated	Sanofi	No	Not stated
Huntington Society of Canada	$100 000-$249 999	2019	Roche	Huntington Society listed as recipient of grant of $71 612	2019
Lung Health Foundation	$100 000-$199 999	2019-2020	AstraZeneca	No	Not stated
Lung Health Foundation	$100 000-$199 999	2019-2020	Boehringer Ingelheim	No	Not stated
Lung Health Foundation	$50 000-$74 999	2019-2020	Bristol Myers Squibb	No	Not stated
Lung Health Foundation	$100 000-$199 999	2019-2020	GlaxoSmithKline	Lung Health Foundation listed as recipient of grant of $67 500	2020
Lung Health Foundation	$50 000-$74 999	2019-2020	Merck	No	Not stated
Lung Health Foundation	$100 000-$199 999	2019-2020	Pfizer	No	2018
Lung Health Foundation	$50 000-$74 999	2019-2020	Sanofi	No	Not stated
Lung Health Foundation	$100 000-$199 999	2019-2020	Takeda	No	Not stated
Obesity Canada	>$100 000	2019	Novo Nordisk	Obesity Canada listed as recipient of grant, amount not given	Not stated

Note: Donations from Innovative Medicines Canada not included.

## Discussion

 Only a quarter of IMC members (11/44) actually reported making donations to patient groups on their websites and frequently only the names of the patient groups were given and the value of the donation and its purpose were omitted. However, patient groups reported receiving donations from an additional 19 IMC members. In addition, some companies made large numbers of donations that were not reported. Therefore, it appears that most companies that made donations were not abiding by the guideline laid out in the IMC Code of Ethical Practices. There were no reports on the IMC website about companies failing to comply with the guideline, so it also appears that the organization is not proactively monitoring this part of its code. (Rx&D, the previous name of IMC, said that the provision was a guideline and not a requirement because Rx&D “could not enforce the terms of a company’s relationship with a patient organization if that group was not prepared to be fully transparent”^8^). Further, although IMC recommends that companies report donations, that requirement does not appear to apply to IMC itself.

 Patient groups reported receiving 373 donations from IMC donors, but almost one-third (35/114) did not report receiving donations from companies that claimed that they donated to the patient groups. Patient groups also infrequently reported the year the donations were made (26.8%) and no patient group reported the exact value of the donation, although 22.0% reported the dollar range of the donation. Only 4 groups reported the purpose of the donations.

 Underreporting of donations by patient groups may be due to a number of factors; companies may channel donations to patient groups through nonprofit entities that they control or substantially fund or through umbrella organizations that are not readily identifiable with those companies.^9^ Groups may also be concerned that publicly identifying donors could undermine their credibility in advocating for their membership.

 Reporting donations by dollar ranges masks the actual amount given and can create a false sense of transparency. For example, Bayer’s choice not to indicate the amount of the donation but rather to say that donations were for greater than $5000 tells us very little about how much Bayer actually donates to patient groups. None of the 79 patient groups that reported receiving donations also reported on the percent of their overall revenue represented by donations or gave information to make that calculation. The frequent absence of dates of donations by both companies and patient groups means that reporting is not transparent and also contributes to the difficulty in linking the donations that companies give with the donations that patient groups receive even for relatively large donations. Information of this nature is necessary in order to determine if there is an association between the level of industry funding and the positions that patient groups take when the industry interests may be affected. A recent systematic review of the literature on industry funding of patient groups identified four studies that investigated the association between patient group funding and positions that these groups took and concluded that industry funded groups tended to have positions favourable to the sponsor.^10^

 The results of this research are broadly in line with studies in other countries. A systematic review that included five studies examining the financial relationship between patient organizations and the health industry found that the median proportion of organizations receiving industry funding and acknowledging it was 29%.^11^ Drug companies reported providing funding to 157 Italian patient groups, but only 46 of these groups named at least one pharmaceutical company as providing funds. Of those 46, three (6.5%) reported the amount of funding, 25 (54.4%) the activities funded and none the proportion of income derived from drug companies.^12^ In Australia, 52.3% of patient groups acknowledged that they had received company donations and of that group only 52.9% of patient groups reported the use of the donations that they received, 13.2% the amount and 4.4% the proportion of their income that came from industry.^13^ Only 25% of the American health advocacy organizations that received grants from Eli Lilly acknowledged Lilly’s contributions on their websites.^14^ The percent of the membership of the Association of the British Pharmaceutical Industry that reported making donations to patient groups varied from 45% to 66% depending on the year.^15^ In the United Kingdom, 63 companies reported payments to patient groups, but 84 companies were mentioned by recipients. Although donors listed 425 recipients, only 200 (47.1%) reported receiving payments. The number and value of payments reported by donors were 259.8% and 163.7% greater than those reported by recipients, respectively.^16^

 Differences in companies’ disclosures about the donations that they make to patient groups may be a reflection of differences in jurisdictional requirements. The codes from the Association of the British Pharmaceutical Industry^17^ and the European Federation of Pharmaceutical Industries and Associations^18^ oblige member companies to publicly disclose a list of patient organizations to which they provide donations. Similarly, each member company in Medicines Australia must list health consumer organisations to which it provides financial support and Medicines Australia publishes the reports on its publicly available website.^19^ In contrast, PhRMA (Pharmaceutical Research and Manufacturers of America), the American lobby group, does not require member companies to report on which patient groups have received donations from them.^20^

###  Limitations

 No attempt was made to compare the total and relative number of donations made by companies and received by patient groups and therefore no “joint” list of donations could be constructed for a number of reasons. The year when donations were made and received was not regularly reported. Therefore, the lack of congruence between reporting of donations made and received could have been because a company made a donation in 2019 but it was not reported by the patient group since the group was reporting donations for 2020. Some companies or groups may keep reports for only one year, meaning some payments made more than a year ago become “invisible,” but other companies or groups may keep them for longer. Also, some companies and groups may report per calendar year; others, soon after the funded activity has started or ended. Even if donations were reported by companies and groups for the same year, the lack of detail about the size of the donations means that there is no guarantee that it was the same donation. The lack of standardization in the website location of statements about donations given and received may have resulted in some donations being missed, although an attempt was made to avoid this problem by using Google to search on companies’ and patient groups’ websites. The results of this study only apply to companies that are members of IMC and reporting of donations may be different for other companies. The 164 donations that IMC members reported making is an underestimate, due to under-reporting by companies. Similarly, there was underreporting by patient groups, so the exact number of donations that they collectively received from IMC members is not known. The patient groups identified in this study is not a comprehensive list of those in Canada. For example, the paper by Lexchin^1^ listed an additional 45 groups not included here. Finally, the data was only extracted by a single person, possibly leading to errors in recording the information, but these errors would have only been in transcribing data since no value judgements were involved. Minor transcription errors, if they occurred, would not alter the overall findings from this research.

## Conclusion

 Reporting on websites about donations by both companies and patient groups in Canada is haphazard, inconsistent and incomplete at present. In order to remedy this situation, patient groups should be required to post annual reports containing specified information on each donation: the name of the company making the donation, the purpose of the donation, the exact amount of the donation and the percent of their annual income that each individual donation represents and the year of the donation. All of this information should be posted under the same heading on all patient groups’ websites. Based on the experience in the United Kingdom, voluntary reporting of industry donations on the websites of charity regulators results in significant under-reporting.^16^ This finding argues for mandatory reporting by patient groups, possibly as a requirement for the retention of their charitable status.

 IMC should change its guideline about reporting donations into a requirement of membership in the organization and proactively enforce the relevant section of its Code. In addition, it should specify the information that companies must provide, including purpose, exact amount of each individual donation and the year of the donation. IMC should also require reporting to be done in the same location on all company websites to simplify locating disclosures about donations. Access to information about donations would be greatly simplified if IMC collated all of the information from individual companies and published it annually on its own website. If companies do not make full and complete reports, IMC should impose sanctions as allowed for in its Code. Finally, IMC itself should be subject to these Code requirements.

 However, even if IMC undertakes these changes, its Code will not apply to non-members, such as the 56 companies that made donations to patient groups in this study. In the United Kingdom, the majority of companies failing to disclose payments were not members of the Association of the British Pharmaceutical Industry although most had voluntarily agreed to abide by its code.^21^ Before the Ontario election in 2019, the government was finalizing regulations for Bill 160 which required that all drug and device manufacturers that provided a “transfer of value” to individual healthcare practitioners and healthcare organizations, including patient groups, report those transfers to a public registry.^22^ The legislative process stopped when the government changed post-election. The federal government should pass similar legislation to make reporting mandatory on a national basis, but until that happens the Ontario government should complete the regulations so that the provincial legislation can come into effect.

 Providing more information about donations will increase the accountability of both companies and patient groups and heighten transparency about their activities.

## Acknowledgements

 Sharon Batt, Alice Fabbri, Adriane Fugh-Berman, Barbara Mintzes, Lisa Parker and Adrienne Shnier provided valuable comments on a draft of the manuscript.

## Ethical issues

 All of the data for this study was publicly available and ethics approval was not required.

## Competing interests

 In 2018-2021, JL received payments for writing a brief in an action for side effects of a drug for Michael F. Smith, Lawyer and a second brief on the role of promotion in generating prescriptions for Goodmans LLP and from the Canadian Institutes of Health Research for presenting at a workshop on conflict-of-interest in clinical practice guidelines. He is currently a member of research groups that are receiving money from the Canadian Institutes of Health Research and the Australian National Health and Medical Research Council. He is a member of the Foundation Board of Health Action International and the Board of Canadian Doctors for Medicare. He receives royalties from University of Toronto Press and James Lorimer & Co. Ltd. for books he has written.

## Author’s contribution

 JL is the single author of the paper.

## Supplementary files


Supplementary file 1. Innovative Medicines Canada Members Not Reporting Making Any Donations to Patient Groups.

Supplementary file 2. Patient Groups Reporting Receiving Donations From Innovative Medicines Canada Member Companies.

Supplementary file 3. Patient Groups Not Reporting Receiving Any Donations From Innovative Medicines Canada Members.
