# Supplementary material for: Donations Made and Received: A Study of Disclosure Practices of Pharmaceutical Companies and Patient Groups in Canada
Source: Int J Health Policy Manag. 2021 Dec 14;11(10):2046–53. doi: 10.34172/ijhpm.2021.172 (PMC9808287; doi:10.34172/ijhpm.2021.172)
Supplement: Supplementary file 1 — Innovative Medicines Canada Members Not Reporting Making Any Donations to Patient Groups. [file ijhpm-11-2046-s001.pdf]

Article title: Donations Made and Received: A Study of Disclosure Practices of Pharmaceutical Companies and Patient Groups in Canada

Journal name: International Journal of Health Policy and Management (IJHPM)

Authors' information: Joel Lexchin[1,2,3*]

[1]School of Health Policy and Management, York University, Toronto, ON, Canada.

[2]University Health Network, Toronto, ON, Canada.

[3]Faculty of Medicine, University of Toronto, Toronto, ON, Canada.

(*Corresponding author: jlexchin@yorku.ca)

Supplementary file 1. Innovative Medicines Canada Members Not Reporting Making Any Donations to Patient Groups

Akcea Therapeutics, Amgen*, Astellas*, AstraZeneca*, BioVectra, Boehringer Ingelheim*, Bristol-Myers Squibb*, Brunel, Ceapro, Charles River Laboratories*, EMD Serono*, Endoceutics, Gilead*, Horizon Therapeutics*, Incyte, Ipsen*, Janssen*, JN Nova, Knight*, Lilly*, Lundbeck, Medicago, Merck*, Nordic Pharma, Otsuka, Ropack Pharma Solutions, Sanofi*, Santen, Servier*, Sobi*, Sunovion*, Thera Technologies, Vantage Biotrials

*Patient groups reported receiving donations from company
