# Supplementary material for: Donations Made and Received: A Study of Disclosure Practices of Pharmaceutical Companies and Patient Groups in Canada
Source: Int J Health Policy Manag. 2021 Dec 14;11(10):2046–53. doi: 10.34172/ijhpm.2021.172 (PMC9808287; doi:10.34172/ijhpm.2021.172)
Supplement: Supplementary file 2 — Patient Groups Reporting Receiving Donations From Innovative Medicines Canada Member Companies. [file ijhpm-11-2046-s002.pdf]

Article title: Donations Made and Received: A Study of Disclosure Practices of Pharmaceutical Companies and Patient Groups in Canada

Journal name: International Journal of Health Policy and Management (IJHPM)

Authors' information: Joel Lexchin[1,2,3*]

[1]School of Health Policy and Management, York University, Toronto, ON, Canada.

[2]University Health Network, Toronto, ON, Canada.

[3]Faculty of Medicine, University of Toronto, Toronto, ON, Canada.

(*Corresponding author: jlexchin@yorku.ca)

Supplementary file 2. Patient Groups Reporting Receiving Donations From Innovative Medicines Canada Member Companies

Name of patient group	Number of donations received	Year donations received	Amount of donation	Percent of overall patient group income from donations	Purpose of donation	Pathway from home page of patient group to list of companies making donations (number of mouse clicks)
Acromegaly Ottawa Awareness and Support Network	3	Not stated	Not stated	Not stated	Not stated	Sponsors (1)
Action Hepatitis Canada	3	Not stated	Not stated	Not stated	Not stated	Sponsors (1)
Arthritis Alliance of Canada	7	2019	Not stated	Not stated	Not stated	About AAC: About AAC executive summary (2)
Arthritis Society	9	Not stated	Not stated	Not stated	Not stated	About us: Partners (2)
Asthma Canada	5	Not stated	Not stated	Not stated	Not stated	Get involved: Our partners (2)
bchepatitis network	2	Not stated	Not stated	Not stated	Not stated	About us: Who we are (2)
Bladder Cancer Canada	5	Not stated	Not stated	Not stated	Not stated	About us: Sponsors & partners (2)
Cactus Montreal	1	Not stated	Not stated	Not stated	Not stated	Actions: Partnerships (2)
CADDAC (Centre for ADHD Awareness, Canada)	3	2020	Not stated	Not stated	Not stated	About CADDAC: Funding (2)
Canadian Arthritis Patient Alliance	1	Not stated	Not stated	Not stated	Not stated	About us (1)
Canadian Association of Psoriasis Patients	5	July 1, 2018-June 30, 2019	Not stated	Not stated	Not stated	About us: CAPP annual report 2018-2019 (2)
Canadian Breast Cancer Network	9	Not stated	Not stated	Not stated	Not stated	About CBCN: Our supporters (2)
Canadian Cancer Society	7	Not stated	Dollar range	Not stated	Not stated	Get involved: Partnerships: Our corporate partners (3)
Canadian Cancer Survivor Network	13	2017	Not stated	Not stated	Not stated	About us: Annual reports: 2017 annual report (3)

Canadian Council for the Blind	3	2021	Category†	Not stated	Not stated	Publications: White Cane magazine: White Cane magazine in pdf format (3)
Canadian Digestive Health Foundation	4	Not stated	Not stated	Not stated	Not stated	Home page (0)
Canadian Hemophilia Society	6	Not stated	Dollar range	Not stated	Not stated	About the CHS: Corporate philanthropy (2)
Canadian Hemophilia Society - Quebec Chapter	5	2019	Not stated	Not stated	Not stated	Provincial chapters: Quebec: Communications: Annual report: Rapport annuel 2019 (5)
Canadian Liver Foundation	8	Not stated	Not stated	Not stated	Not stated	We are liver: Partners: Corporate partners (3)
Canadian National Institute for the Blind	2	2020-2021	Dollar range	Not stated	Not stated	Support us: Partner: National corporate and foundation partners (2)
Canadian Organization for Rare Diseases	7	2020-2021	Not stated	Not stated	Not stated	CORD membership: CORD corporate leaders (2)
Canadian Psoriasis Network	7	Not stated	Not stated	Not stated	Not stated	Home page (0)
Canadian Pulmonary Fibrosis Foundation	2	Not stated	Category	Not stated	Not stated	About CFPP: Sponsors (2)
Canadian Skin Patient Alliance	8	Not stated	Not stated	Not stated	Not stated	About us: Sponsors (2)
Canadian Spondylitis Association	8	Not stated	Not stated	Not stated	Not stated	Who we are: Corporate sponsors (2)
CAPAHC	3	Not stated	Not stated	Not stated	Not stated	About us (1)
Cassie + Friends	4	Not stated	Not stated	Not stated	Not stated	Support: Virtual education (2)
CATIE	2	Not stated	Dollar range	Not stated	Not stated	About CATIE: Funders and partners (2)
Centre SIDA Amitié	2	Not stated	Not stated	Not stated	Not stated	Home page (0)
Childhood Cancer Canada	1	Not stated	Not stated	Not stated	Not stated	Partners: Corporate sponsors (2)
CNETS - Canadian Neuroendocrine Tumour Society	4	2020	Not stated	Not stated	Not stated	About: Funders (2)
Coalition Priorite Cancer	12	2020	Category	Not stated	Not stated	Members and partners: Partners (2)
Colorectal Cancer Canada	13	Not stated	Not stated	Not stated	Not stated	Learn about us: Prevention (2)
Colorectal Cancer Resource	4	Not stated	Not stated	Not stated	Not stated	Home page (0)
COPD Canada	6	Not stated	Not stated	Not stated	Not stated	Home page (0)
CPBF Canadian Premature Babies Foundation	1	Not stated	Not stated	Not stated	Not stated	About us: Supporters (2)
Crohn’s and Colitis Canada	5	Not stated	Not stated	Not stated	Not stated	Home page (0)
Diabetes Canada	10	Not stated	Dollar range	Not stated	Not stated	Get involved: Corporate partnerships: Corporate recognition (3)
Diabetes Hope Foundation	1	Not stated	Not stated	Not stated	Not stated	About us: Our sponsors (2)

Eczema Society of Canada	6	Not stated	Category	Not stated	Not stated	Get involved: Sponsors and donors (2)
Foundation Fighting Blindness	4	Not stated	Category	Not stated	Not stated	About us: Corporate partners (2)
Gastrointestinal Stroma Tumour (GIST) Sarcoma Life Raft Group Canada	2	Not stated	Not stated	Not stated	Not stated	About us: Our stakeholders (2)
Heart and Stroke Foundation	10	Not stated	Dollar range	Not stated	Not stated	About us: Our partners (2)
HeartLife Foundation	1	Not stated	Not stated	Not stated	Not stated	Home (0)
Help for Headache	6	Not stated	Not stated	Not stated	Not stated	Supporters (1)
Hemophilia Ontario	6	Not stated	Not stated	Not stated	Not stated	About us: Our partners (2)
HepCBC	1	2019-2020	Dollar range	Not stated	Not stated	About us: Sponsors & donors: Sponsors (3)
Huntington Society of Canada	1	2019	Dollar range	Not stated	Not stated	About us: Annual report and financials: Annual report (3)
Kidney Cancer Canada	7	Not stated	Not stated	Not stated	Not stated	About us: Our partners and sponsors (2)
Les Diabétiques de Québec	7	2019-2020	Not stated	Not stated	Not stated	L'association: Rapport d'activités 2019-2020 (2)
Leukemia & Lymphoma Society of Canada	2	Not stated	Not stated	Not stated	Not stated	About: Our partners (2)
Lung Association - Quebec	7	Not stated	Category	Not stated	Not stated	About us: Partners (2)
Lung Cancer Canada	10	2019	Not stated	Not stated	Not stated	About us: Corporate supporters (2)
Lung Health Foundation	9	2019-2020	Dollar range	Not stated	Not stated	About us: Annual reports & financials: 2019-2020 (2)
Lymphoma Canada	7	Not stated	Not stated	Not stated	Not stated	About us: Our partners (2)
Melanoma Network of Canada	5	Not stated	Not stated	Not stated	Not stated	About MNC: MNC corporate partners (2)
Migraine Canada	2	Not stated	Not stated	Not stated	Not stated	Partners (1)
Migraine Quebec	3	Not stated	Not stated	Not stated	Not stated	Home (0)
Muscular Dystrophy Canada	2	2019	Not stated	Not stated	Yes	Services & support: Research: Research new: Key topics in Spinal Muscular Atrophy research discussed at first ever Muscular Dystrophy Canada SMA Research Summit (4)
Myeloma Canada	11	Not stated	Not stated	Not stated	Not stated	Home page (0)
Network of Rare Blood Disorders Organizations	3	Not stated	Not stated	Not stated	Not stated	Home page (0)
Obesity Canada	3	2019	Dollar range	Not stated	Not stated	About us: Partners (2)
Parkinson Alberta	1	Not stated	Not stated	Not stated	Not stated	Home page (0)
Parkinson Canada	1	Not stated	Not stated	Not stated	Yes	Get involved: Current priorities: Include your voice (3)

Parkinson Quebec	3	Not stated	Dollar range	Not stated	Not stated	About us: Our partners (2)
Parkinson Society British Columbia	2	2019	Dollar range	Not stated	Not stated	About us: Annual reports: Annual report 2019 (3)
Parkinson Society Southwestern Ontario	1	2021	Not stated	Not stated	Yes	Events: Parkinson's conference (2)
Platelet Disorder Support Association	2	Not stated	Category	Not stated	Not stated	Home (0)
Préma-Québec	1	Not stated	Not stated	Not stated	Not stated	About us: Our partners (2)
Procure	4	2017	Dollar range	Not stated	Not stated	Learn more: What we do: Reports and brochures: Annual reports (4)
PROSTAID CALGARY - Prostate Cancer Support	3	Not stated	Not stated	Not stated	Not stated	Donate: Sponsors and donors (2)
Pulmonary Association of Quebec	8	Not stated	Category	Not stated	Not stated	About us: The association: Partners (2)
Quebec Breast Cancer Foundation	4	Not stated	Dollar range	Not stated	Not stated	Our foundation: Partners (2)
Rethink Breast Cancer	6	Not stated	Not stated	Not stated	Not stated	Support our movement: Partner with us (2)
Robbie's Rainbow	1	Not stated	Not stated	Not stated	Yes	Resources: Education guides: Preparing for your clinic appointments (3)
Save Your Skin Foundation	11	Not stated	Not stated	Not stated	Not stated	Get involved: Thanks to our sponsors (2)
Sickle Cell Awareness Group of Ontario	2	Not stated	Not stated	Not stated	Not stated	Home (0)
Thrombosis Canada	6	Not stated	Not stated	Not stated	Not stated	About us: Partners (2)
Vivre 100 Fibromes	1	Not stated	Not stated	Not stated	Not stated	Home page (0)

*Grants from Innovative Medicines Canada not included

†Category, e.g., gold, silver, bronze donation
