# Supplementary material for: Donations Made and Received: A Study of Disclosure Practices of Pharmaceutical Companies and Patient Groups in Canada
Source: Int J Health Policy Manag. 2021 Dec 14;11(10):2046–53. doi: 10.34172/ijhpm.2021.172 (PMC9808287; doi:10.34172/ijhpm.2021.172)
Supplement: Supplementary file 3 — Patient Groups Not Reporting Receiving Any Donations From Innovative Medicines Canada Members. [file ijhpm-11-2046-s003.pdf]

Article title: Donations Made and Received: A Study of Disclosure Practices of Pharmaceutical Companies and Patient Groups in Canada

Journal name: International Journal of Health Policy and Management (IJHPM)

Authors' information: Joel Lexchin[1,2,3*]

[1]School of Health Policy and Management, York University, Toronto, ON, Canada.

[2]University Health Network, Toronto, ON, Canada.

[3]Faculty of Medicine, University of Toronto, Toronto, ON, Canada.

(*Corresponding author: jlexchin@yorku.ca)

Supplementary file 3. Patient Groups Not Reporting Receiving Any Donations From Innovative Medicines Canada Members

AAMAC (Aplastic Anemia & Myelodysplastic Association of Canada), ADHD Awareness Windsor, AIDS Committee of Ottawa, Arthritis Consumer Experts, Balance for Blind Adults, BloodTies, BRAS, Canadian Headache Society, Canadian MPN Network, Canadian MPN Research Foundation, Cancer Collaborative, Cancertainty, CLL Canada, Colorectal Cancer Association, Dopamine, GI Society (Canadian Society of Intestinal Research), HepNS, Kidney Foundation, Life with a Baby, Lung Association - British Columbia, Lung Association – Manitoba, Lung Association - New Brunswick, Lung Association - Nova Scotia, Lupus Canada, MS Society of Canada, MS Society of Canada – Quebec Division, Ovarian Cancer Canada, Pulmonary Hypertension Association of Canada, Quebec Association for Macular Degeneration, Sarcoma Cancer Foundation of Canada, Sickle Cell Disease Association of Canada, Stop Diabetes Foundation, Thalassemia Foundation of Canada, The Canadian CML Network, Vancouver Acromegaly Support Group
